# Inflammatory nociception diminishes dopamine release and increases dopamine D2 receptor mRNA in the rat's insular cortex

**DOI:** 10.1186/1744-8069-6-75

**Published:** 2010-11-04

**Authors:** Ulises Coffeen, J Manuel Ortega-Legaspi, Patricia de Gortari, Karina Simón-Arceo, Orlando Jaimes, María Isabel Amaya, Francisco Pellicer

**Affiliations:** 1 Laboratorio de Neurofisiología Integrativa, Dirección de Neurociencias, Instituto Nacional de Psiquiatría Ramón de la Fuente, México; 2 Laboratorio de Neurofisiología Molecular, Dirección de Neurociencias, Instituto Nacional de Psiquiatría Ramón de la Fuente, México; 3 Emory University School of Medicine Atlanta Georgia, USA

## Abstract

**Background:**

The insular cortex (IC) receives somatosensory afferent input and has been related to nociceptive input. It has dopaminergic terminals and D1 (D1R) -excitatory- and D2 (D2R) -inhibitory- receptors. D2R activation with a selective agonist, as well as D1R blockade with antagonists in the IC, diminish neuropathic nociception in a nerve transection model. An intraplantar injection of carrageenan and acute thermonociception (plantar test) were performed to measure the response to inflammation (paw withdrawal latency, PWL). Simultaneously, a freely moving microdyalisis technique and HPLC were used to measure the release of dopamine and its metabolites in the IC. Plantar test was applied prior, one and three hours after inflammation. Also, mRNA levels of D1 and D2R's were measured in the IC after three hours of inflammation.

**Results:**

The results showed a gradual decrease in the release of dopamine, Dopac and HVA after inflammation. The decrease correlates with a decrease in PWL. D2R's increased their mRNA expression compared to the controls. In regard of D1R's, there was a decrease in their mRNA levels compared to the controls.

**Conclusions:**

Our results showed that the decreased extracellular levels of dopamine induced by inflammation correlated with the level of pain-related behaviour. These results also showed the increase in dopaminergic mediated inhibition by an increase in D2R's and a decrease in D1R's mRNA. There is a possible differential mechanism regarding the regulation of excitatory and inhibitory dopaminergic receptors triggered by inflammation.

## Background

The involvement of dopamine in the supraspinal modulation of pain processes has been widely described [1,2]. This evidence was first provided, in the clinical field, by patients with Parkinson's disease who among other symptoms experience an altered pain perception [3]. Also, patients with diabetic polyneuropathy who had administration of L-dopa showed a decrease in pain [4]. Moreover, the role of dopamine D2 receptors became evident in the striatal binding capacity associated with response to cold pain [5].

Analogous to human research, evidence from animal studies indicates that the supraspinal dopaminergic system is widely involved in pain processing. Thus, the dopaminergic neurones in the nucleus accumbens are related with the suppression of tonic pain [6]. Furthermore, the electrical stimulation of the ventral tegmental area, which is a dopaminergic nucleus that projects to several pain matrix structures, diminishes pain related behaviour [7-9]. Also, the local administration of dopamine or its agonists in pain matrix structures such as the striatum, nucleus accumbens, anterior cingulate, ventrolateral orbitofrontal and insular cortices diminishes pain related behaviours [10-15].

The insular cortex (IC) as a component of the limbic system and the pain matrix is involved in the dopaminergic modulation of nociception. The use of a dopamine uptake inhibitor in the IC increases the thermonociceptive threshold [14]. Also, there are dopamine D1 and D2 receptors in the IC [16,17]. D2 receptor activation and D1 blockade diminish neuropathic pain related behaviour [18]. This suggests that there is a differential modulation of dopaminergic receptors in the IC in relation to chronic nociceptive processes.

Given the relationship between dopamine, its receptors and nociceptive modulation in the IC, we decided to measure the release of dopamine and its metabolites (DOPAC and HVA) as well as dopamine D1 and D2 receptors' mRNA expression. Both measurements were done in the IC during the development of an inflammatory pain model in the rat.

## Results

### Dopamine release in the insular cortex

In this experiment we measured extracellular dopamine release in the IC during an inflammatory process induced by the intraplantar injection of carrageenan. Simultaneously, the behavioural thermonociceptive response (paw withdrawal latency, PWL, to thermal nociceptive stimulation) was measured prior to inflammation, as well as one and three hours afterwards.

**Figure 1:**
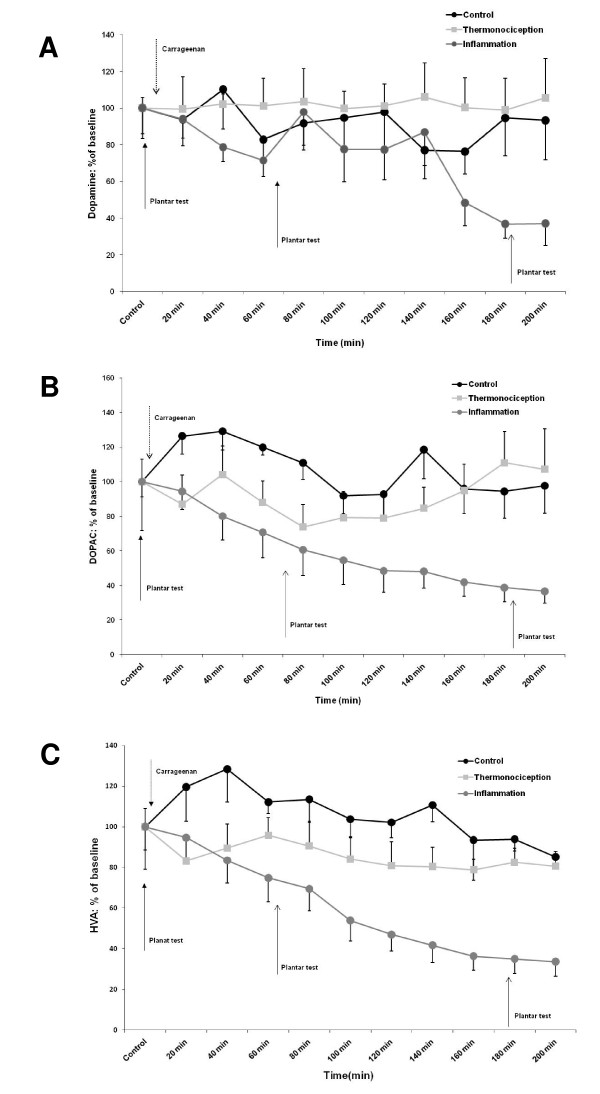
**The graphs show extracellular levels of (A) dopamine, (B) DOPAC and (C) HVA in the insular cortex**. The data are expressed as the percentage of their respective basal levels. Notice that group with inflammation (which received an intraplantar injection of carrageenan) shows a decreased percentage in the levels of dopamine and its metabolites when compared with the control and thermonociception groups. Repeated measures ANOVA, Dopamine (F = 7.817 p = 0.021, n = 6), DOPAC (F = 11.223 p = 0.002, n = 8) and HVA (F = 6.902 p = 0.006, n = 8).

The release of dopamine and its metabolites in the inflammation group was decreased when compared to control and thermonociception groups. This decrease was of 28.6% 1 h after the induction of inflammation with carrageenan (p < 0.05) and 63.2% after 3 h for dopamine (p < 0.05). Regarding DOPAC's, it decreased 29.4% at 1 h (p < 0.05) and 61.4% at 3 h (p < 0.001). Similarly, HVA lowered its value 25.2% at 1 h (p < 0.05) and 65% at 3 h (p < 0.05; Figure 1).

**Figure 2:**
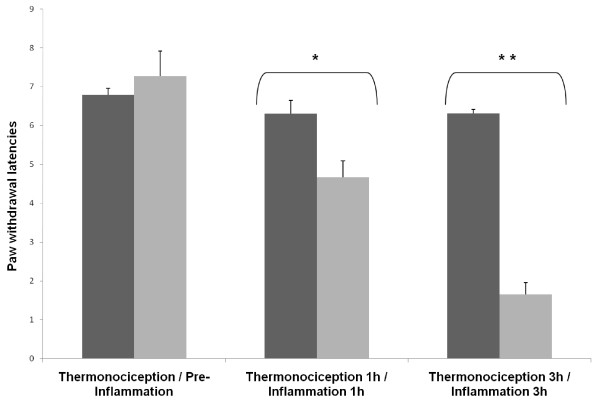
**Antialgesic nociceptive response in the inflammation and thermonociception groups measured as paw withdrawal latency (PWL) in s after a thermonociceptive stimulus**. The figure shows PWL before the induction of inflammation with carrageenan 1% (250 μl) in the inflammation group in the first set of bars. The second and third sets of bars show PWL 1 h and 3 h after inflammation, respectively. The thermonociception groups did not have inflammation induced and were tested in parallel with the inflammation group. Notice that the inflammation group has no difference in PWL before inflammation compared to the thermonociception group and shows a progressively decreased PWL 1 h (t = -2.335, p = 0.035) and 3 h (t = -9.938, p < 0.001) thereafter.

In regard of PWL in this experiment, there was a decrease of 27% at 1 h (t = -2.335, P = 0.035) and 74.7% at 3 h (t = -9.938, P < 0.001) after inflammation in the inflammation group compared with control and thermonociception groups (Figure 2).

**Figure 3:**
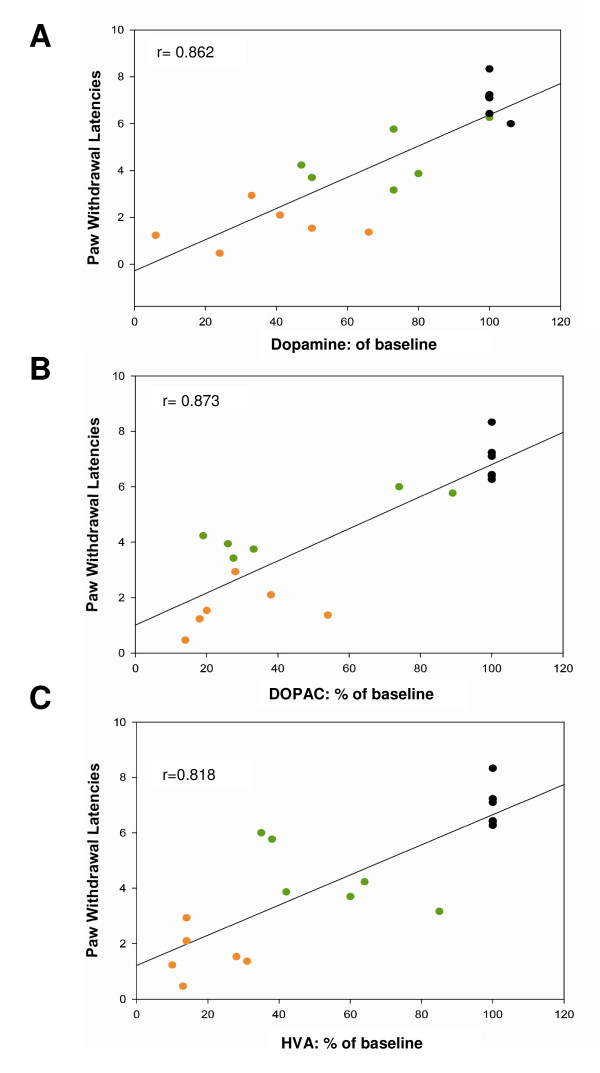
**The figure represents a positive correlation between paw withdrawal latency (PWL) and (A) dopamine, (B) DOPAC or (C) HVA**. The dots in orange depict the measurements three hours after the carrageenan injection, the green ones 1 h afterwards and the black ones show the status prior to inflammation. Notice that as the release of dopamine and its metabolites increases, so does PWL and vice versa (Pearson correlation, dopamine r = 0.862, p < 0.001; DOPAC r = 0.873, p < 0.001; and HVA r = 0.818, p < 0.001).

Moreover, there was a positive correlation between the decrease in dopamine, DOPAC and HVA release in the IC with the decrease in PWL in the inflammation groups (Pearson correlation: dopamine r = 0.862, p < 0.001; DOPAC r = 0.873, p < 0.001; HVA r = 0.818, p < 0.001) (Figure 3).

### mRNA levels of dopamine D1 and D2 receptors in the IC

This experiment evaluated if the same inflammatory process as above could produce changes in the expression of dopamine D1 and D2 receptors' mRNA levels in the IC.

**Figure 4:**
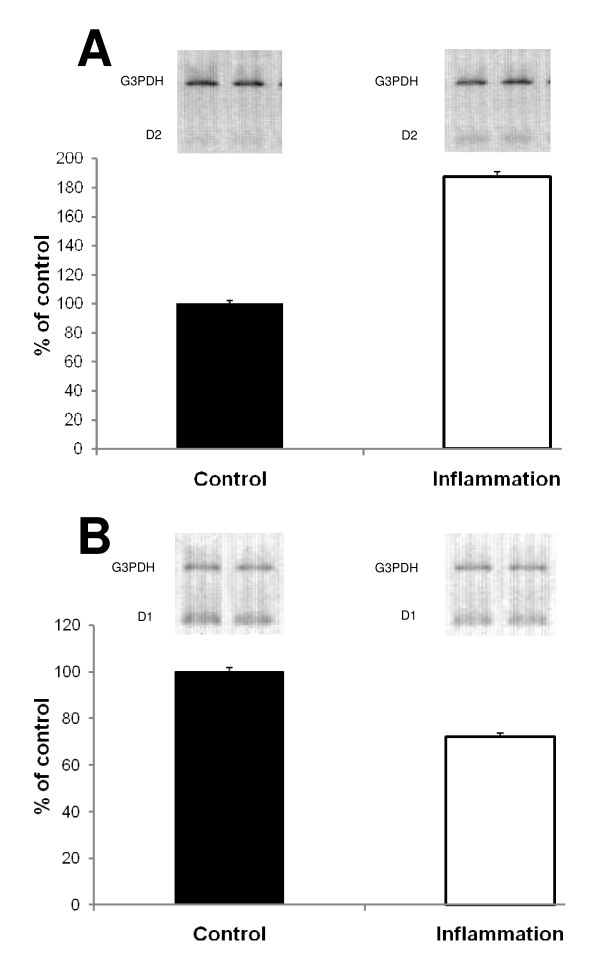
**D1 and D2 mRNA levels were semi-quantified by RT-PCR and related to G**_**3**_**PDH mRNA**. Values are the ratio of D1 or D2 cDNA/G_3_PDH and expressed as the mean ± SEM calculated as % of control (thermonociception) control values (C = 100%). Frame (A) shows an increase in dopamine D2 receptor mRNA levels in the group with inflammation (188 ± 3.2%) with respect to 100% of the control (p = 0.000). Frame (B) shows a decrease in dopamine D1 receptor mRNA levels in the group with inflammation (72 ± 1.6%) with respect to the control (100%, p = 0.000). At the top of each bar, there is an example of the electrophoresis agarose gel showing the control oligonucleotide (upper band) and the cDNA of D1R's in (B) and D2R's in (A) (lower band).

Dopamine D1 receptor mRNA showed a decrease of 28% (t = -11.167, P = <0.001) in the inflammation group compared to the control. As opposed to this result, mRNA levels of D2R were increased in the inflammation group by 88% (t = -22.468, P < 0.001) (Figure 4).

## Discussion

The insular cortex is a well known locus of somatosensory input convergence in humans. Functional imaging studies consistently show that it is activated after nociceptive input and that it is involved in cognitive and affective component related with empathy and anticipation of pain [19-24]. Similar evidence is also available in the rat, in which painful stimulation activates the equivalent structure to the human insular cortex [25-28].

This study highlights the dopaminergic processes involved in the modulation of peripheral inflammatory pain in the insular cortex. The results showed that extracellular level of dopamine decrease and D2 receptor mRNA levels increase, both correlating with a decrease in paw withdrawal latency. Interestingly, dopamine levels do not vary after repetitive acute thermonociception. This suggests the role played by the insular cortex in long lasting pain processes. These processes are subject of being modulated in supraspinal structures rather than acute ones which are mainly integrated in the spinal cord [29].

In contrast, Gao and colleagues [30] found that dopamine levels increase in the striatum, periaqueductal grey and spinal dorsal horn after subjecting animals to the same model of nociception. Regarding the results found in the IC, it is a pro-nociceptive locus which when stimulated with dopaminergic agonists produces a decrease in pain related behaviours [14,18]. Therefore, since it ought to be activated after nociceptive input, physiological release of dopamine would need to be reduced. Neuroanatomical evidence that supports this suggestion is given by the evidence in which a lesion is performed in the IC. After the animals were subjected to several nociceptive tests, all the behaviours were diminished [31]. Moreover, if dopamine D1 receptors are blocked in the IC, chronic pain related behaviour is also decreased [18].

The insular cortex expresses a higher quantity of excitatory dopamine D1 receptors [16,17]. Therefore, in this work a double compensatory mechanism becomes evident. On the one hand, there is a decrease in dopamine release and D1 receptors' mRNA levels and on the other, an increase in inhibitory dopamine D2 receptors' expression. Interestingly, both of the aforementioned results correlate with the level of nociceptive behaviour which we measured as PWL. Similar findings were described in the anterior cingulate cortex with the expression of mRNA of dopaminergic D1 and D2 receptors [32]. This suggests an important receptor dynamic in which there is regulation and crosstalk between excitatory and inhibitory receptors in several limbic structures related to pain modulation.

## Conclusions

This study shows evidence about the cellular and dynamic neurotransmitter response in a specific site of the pain matrix after a very frequent event in pain patients, inflammation.

### Material and Methods

The experiments were conducted in agreement with the ethics committee regulations of the International Association for the Study of Pain [33] and with our institution project's and bioethics commission approval.

Male Wistar rats (250-300 g) were raised, housed and maintained in our institution's animal house. The animals were kept in transparent acrylic individual cages, with light-dark cycles of 12:12 h at 23°C and 52% humidity, and with *ad libitum *feeding and hydration.

### Dopamine release in the insular cortex

Extracellular concentration of dopamine and its metabolites were measured in the IC by means of microdialysis in freely moving rats and high performance liquid chromatography (HPLC) during the development of an inflammatory pain model.

### Inflammatory induction and thermal stimulation

An inflammatory process was induced by the infiltration in the right hind paw of carrageenan lambda (Sigma Chemical Co. St. Louis MO, USA, CAR 1% in saline solution, 250 μl).

Thermonociceptive response was measured in a Plantar Test Apparatus (Ugo Basile mod.7370). Paw withdrawal latency (PWL) was determined to the nearest 0.1 second by the electronic clock of the device.

### In vivo microdialysis

Rats were anaesthetised with isofluorane 2% mixed with 98% O_2 _and mounted in a stereotaxic frame. A guide cannula (CMA-11-Microdialysis, Acton, MA) was stereotaxically implanted into the insular cortex. Forty eight hours after cannulation, a microdialysis probe (SciPro Inc. 12, 2 mm tip length) was inserted into the guide cannula so that its tip ended in the IC (A = 1 mm from bregma, L = 4.8 mm, H = 5.8 mm from meninges) using coordinates according to the atlas by Paxinos and Watson (1998). Each probe was continuously perfused at 2 μl/min with sterile artificial cerebrospinal fluid (aCSF) (145 mM NaCl, 2.8 mM KCl, 3.0 mM CaCl2, 5.4 mM D-glucose, pH 7.2) using a microinfusion pump (KD Scientific, Holliston, MA, USA). Animals were individually housed for the duration of the experiment in a system for freely-moving animals and microdialysate samples were collected at 20 min intervals into microvials containing 4 μl of L-Glutathione 0.08% (Sigma-Aldrich) to reduce oxidation of monoamines.

### Biochemical conditions (HPLC)

An isocratic, high-performance liquid chromatography-electrochemical detection (HPLC-ECD) assay was used to quantify dopamine, in 20-μl samples of microdialysate. A mobile phase containing, 95% of 12.5 mM citric acid, 0.07 mM 1-octanesulfonic acid sodium salt, 0.05 mM EDTA and 25 nM ortho phosphoric acid, and 5% of methanol HPLC (adjusted to pH 3.2 with KOH 10 M), was pumped at 0.1 ml/min through an X Terra C 18 (2.1 × 50 mm, 3.5 μm ODS) column. Online data capture was performed using Waters Empower software for HPLC.

The animals were divided in the following groups:

Control (n = 6): basal extracellular concentration of dopamine, DOPAC (d-hydroxy-phenyl acetic acid) and HVA (homovanilic acid) during three hours.

Thermonociception group (n = 6): extracellular concentrations of dopamine, DOPAC and HVA were measured during three hours and plantar test was carried out at the beginning of the microdialysis, and also one and three h later.

Inflammation group (n = 6): extracellular concentrations of dopamine, DOPAC and HVA were measured prior to the induction of inflammation and until three h after its induction. Plantar test was carried out prior to inflammation and one and three h afterwards.

Statistically significant differences (p < 0.05) between groups on extracellular concentrations of dopamine in the IC, were established by repeated measures ANOVA, with a *post hoc *Tukey test.

In order to establish differences in PWL between the control and each experimental group a Student's t-test was performed. Significance was considered with a value of p < 0.05. A Pearson's correlation was performed in order to test if there was a relationship between de dopamine release and PWL.

#### Histological verification

**Figure 5:**
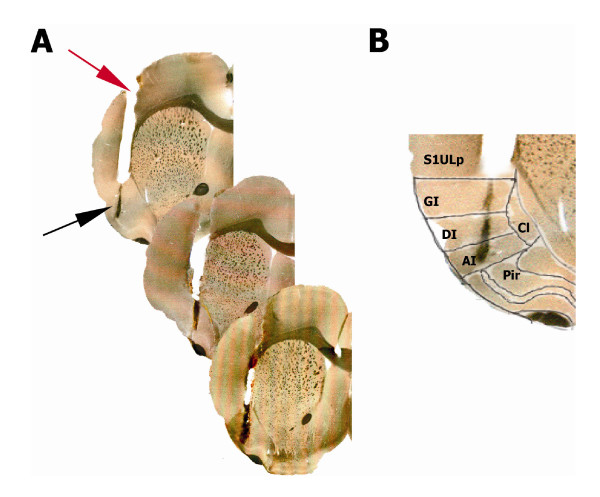
**Histological verification of the microdialysis cannula**. (A) Cannula position in three different experiments. The figures show the guide cannula (red arrow) and membrane trace (black arrow). Notice that the membrane location (black arrow) involves the three regions of the insular cortex (granular, GI; dysgranular, DI; and agranular, AI). (B) A close up of the left IC was superimposed to an image from the atlas by Paxinos and Watson (1998) in order to define the location of the microdialysis probe. The piriform cortex (Pi) is in the ventral border of the IC which corresponds to the AI. The upper limit, corresponding to the GI, is attached to the upper lip region of S1 (S1ULp). The medial border comprising the three regions of the IC corresponds to the claustrum (Cl).

At the end of the experiment the correct microdialysis probe placement was verified. Briefly, the animals were intracardially perfused with physiological saline solution, followed by 10% formaldehyde. The brains were allowed to postfix for 2 days and cut in 40 μm coronal slices that were immediately placed in a glass slide and digitalised in a scanner (HP Scanjet 5550C). The images were analysed by comparing them to an anatomical atlas [34]. (Figure 5)

### Dopamine D1 and D2 mRNA levels in the Insular Cortex

In another series of experiments, dopamine D1 and D2 receptor mRNA levels were analysed in the IC after the induction of an inflammatory process.

### Reverse transcriptase polymerase chain reaction (RT-PCR) procedure

The animals were sacrificed by decapitation and the brain was extracted and frozen in dry ice (-70°C). In order to extract the IC (contralateral to inflamed hind paw), a coronal brain slice was obtained. Then, one punch with a 1.0 mm diameter sample corer was done in the IC [34].

Semi-quantification of mRNA by reverse trascriptase polymerase chain reaction (RT-PCR) was carried out afterwards. Frozen IC was homogenized in 4 M guanidine thyocianate (ICN, Aurora, Ohio, USA) and total RNA extracted as has previously been described [35]. RNA quality of samples was verified by the ratio of O.D. absorbencies 260/280 nm and 260/230 nm considered appropriate when value was >1.8, and by electrophoresis quantifying 28S/18S ratio and discarded if lower than 1.8 or, when evidence of degradation was observed by increased staining at the end of the gel. mRNA levels of D1R and D2R from IC were semi-quantified by reverse-transcriptase polymerase chain reaction (RT-PCR), using glyceraldehyde 3-phosphate dehydrogenase (G3PDH) and actin genes as control transcripts. The protocol used was essentially as described in [36]: 1.5 μg of RNA was used to obtain cDNA (M-MLV reverse transcriptase (Carlsbad, CA, USA) and oligo-dT (Universidad Nacional Autónoma de México UNAM Biotechnology Institute's facilities), followed by the PCR reaction: the number of cycles for each probe was optimized for each region to assure linear conditions, using 1 μl and 25 pmol of D1R probe (sense sequence: CAT TCT GAA CCT CTG CGT GA; antisense: GTT GTC ATC CTC GGT GTC CT), for D2R using 1 μl and 25 pmol (sense sequence: CAT TGT CTG GGT CCT GTC CT; antisense: GAC CAG CAG AGT GAC GAT GA); 1 μl and 50 pmol G3PDH (sense sequence: TGA AGG TCG GTG TCA ACG GAT TTG GC; antisense: CAT GTA GGC CAT GAG GTC CAC CAC); and 1 μl and 25 pmol for actin (sense sequence: GAC GAT GCT CCC CGG GCT GTA TTC; antisense: TCT CTT GCT CTG GGC CTC GTC ACC) and 0.5 μl Taq DNA polymerase (5U/μl) (Biotecnologías Universitarias, UNAM, DF, México). Oligonucleotides were synthesized at the Instituto de Biotecnología, UNAM. Final conditions for the IC were: 29 cycles for D1R, 30 for D2R, 20 for G3PDH and 23 for actin. Each cycle consisted of 95°C for 1 min followed by: 1 min at 55°C for D1R, D2R and 64°C for G3PDH and actin; all followed by 1 min 15 s at 72 °C. All cDNAs had a final extension of 10 min at 72°C. Several cDNAs were semiquantified from the same RT reaction.

PCR products (10 μl of each DNA, and 5 μl of G3PDH) were separated by 2% of agarose (Ultra-pure Bio-Rad, Hercules CA, USA) gel electrophoresis, stained with ethidium bromide (1 mg/L) and density measured with the Advanced American Biotech Imaging software (American-Applied Biotechnology, Fullerton, CA, USA). The relative amounts of the studied cDNAs were calculated as the ratio of each cDNA over G3PDH and actin densities. Care was taken to include samples of controls and experimental groups in the same gel.

There were two groups for this experiment:

Control (n = 8): mRNA was analysed by RT-PCR in animal without interventions.

Inflammation group (n = 8): mRNA was analysed by RT-PCR in rats after three hours of the induction of inflammation.

For the statistical significance between groups a student's t test was used. Significance was considered when p < 0.05.

## Competing interests

The authors declare that they have no competing interests.

## Authors' contributions

UC: carried out the behavioral, HPLC determinations and participated in the design of the study, performed the statistical analysis and drafted the manuscript; JMOL: participated in the design of the study and drafted the manuscript; PG: molecular RT-PCR procedures; KSA: carried out the behavioral procedures; OJ: HPLC determinations; MIA: RT-PCR determinations; FP: conceived of the study, design, coordination and drafted the manuscript. All authors read and approved the final manuscript.
